# Effects of Exosomes Derived From *Helicobacter pylori* Outer Membrane Vesicle-Infected Hepatocytes on Hepatic Stellate Cell Activation and Liver Fibrosis Induction

**DOI:** 10.3389/fcimb.2022.857570

**Published:** 2022-06-27

**Authors:** Masoumeh Ebadi Zahmatkesh, Mariyeh Jahanbakhsh, Negin Hoseini, Saina Shegefti, Amir Peymani, Hossein Dabin, Rasoul Samimi, Shahin Bolori

**Affiliations:** ^1^ Medical Microbiology Research Center, Qazvin University of Medical Sciences, Qazvin, Iran; ^2^ Microbiology Department, Faculty of Medicine, Zanjan University of Medical Sciences, Zanjan, Iran; ^3^ Microbiology Department, Faculty of Medicine, Shahid Beheshti University of Medical Sciences, Tehran, Iran

**Keywords:** *Helicobacter pylori*, outer membrane vesicle, exosome, liver fibrosis, α-SMA

## Abstract

Liver fibrosis is a multifactorial disease with microbial and non-microbial causes. In recent years, *Helicobacter pylori* infection has been thought to play a critical role in some extra-gastrointestinal manifestations especially liver disorders. Outer membrane vesicles (OMVs) are one of the most important discussed *H. pylori* virulence factors. In the current study, four different clinical strains of *H. pylori* were collected and their OMVs were purified using ultra-centrifugation. To investigate their effects on liver cell exosomes, co-incubation with hepatocytes was applied. After a while, hepatocyte-derived exosomes were extracted and incubated with hepatic stellate cells (HSCs) to investigate the HSC activation and fibrosis marker induction. The expression of α-SMA, TIMP-1, β-catenin, vimentin, and e-cadherin messenger RNAs (mRNA) was assessed using real-time RT-PCR, and the protein expression of α-SMA, TIMP-1, β-catenin, vimentin, and e-cadherin was evaluated by Western blotting. Our results showed that infected hepatocyte-derived exosomes induced the expression of α-SMA, TIMP-1, β-catenin, and vimentin in HSCs and e-cadherin gene and protein expression was downregulated. In the current study, we found that *H. pylori*-derived OMVs may aid the exosome alternation and modified exosomes may have a possible role in HSC activation and liver fibrosis progression.

## Introduction

*Helicobacter pylori* is a Gram-negative and microaerophilic bacterium which is able to colonize human gastric (Chmiela et al., 2017). According to the latest epidemiological studies, its prevalence is about 50% of the world population (Mezmale et al., 2020; Ziyaee et al., 2020). *H. pylori* is a causative agent of various diseases including peptic ulcer, chronic gastritis, gastric cancer, and lymphoid malignancies of the stomach (Topi et al., 2020; Kunovsky et al., 2021). A possible role of *H. pylori* pathogenesis in extra-gastric diseases including cardiovascular disease has been studied in previous decades. However, recent studies have highlighted *H. pylori* infection and liver diseases especially non-alcoholic fatty liver disease (NAFLD) ( Buzás, 2020; Lu et al., 2020; Xu et al., 2020). Additionally, the importance of *Helicobacter* species as a risk factor for the progression of liver disease to cirrhosis and hepatocellular carcinoma, especially among patients chronically infected with hepatitis C virus, has been discussed previously (Pellicano et al., 2008). Genetic arrangement and the presence of virulence factors dramatically determine the outcome of *H. pylori* infections. Previous studies showed that CagA and VacA factors are the most crucial virulence factors in *H. pylori* disease progression (Jouimyi et al., 2020; Mubarakali et al., 2021); however, recent studies highlighted the importance of novel virulence factors including outer membrane vesicles (OMVs) (Jarzab et al., 2020; Chew et al., 2021).

Outer membrane vesicles are nano-sized vesicles surrounded by the outer membrane layer that is similar to human membranes (Sartorio et al., 2021). These vesicles contain various factors including potent virulence factors CagA and VacA, different enzymes like HtrA and urease, lipopolysaccharides, and many different factors (Jarzab et al., 2020). Due to their contents, OMVs are thought to play an important role in bacterial pathogenesis, toxin and enzyme delivery, and disease progression (Ansari and Yamaoka, 2019).

Exosomes are one of the most studied extracellular vesicles, budding directly from the cellular membrane (Lee et al., 2012). Studies showed that exosomes are a complex package of different proteins, lipids, polysaccharides, DNAs, and coding and non-coding RNAs (García-Contreras et al., 2015). As is known, the most important role of exosomes is the cell-to-cell communication with their potent factors like miRNA and proteins (Turturici et al., 2014). Moreover, for over a few years exosomes have been studied extensively concerning the pathobiology of NAFLD which was indicated as a key modulator in the setting of immune-mediated inflammation (Srinivas et al., 2021). However, in recent years, studies have highlighted the modification of exosome content by pathogens during infection as a novel mechanism in systemic pathogenesis especially among patients suffering from NAFLD (Fleming et al., 2014; Wang et al., 2018).

According to the importance of *H. pylori*-derived OMVs in pathogenesis, the role of bacterial virulence factors in exosome content modification, and the role of exosomes in cell-to-cell communications and *H. pylori* association with liver diseases, this study aims to investigate the effects of *H. pylori*-derived OMVs on hepatocyte exosome modification and their effects on hepatic stellate cell activation.

## Material and Methods

### Bacterial Culturing, OMV Isolation, and Characterization

Four different *H. pylori* strains were obtained from the Medical Microbiology Research Center, Qazvin, Iran. Strains were routinely cultured in *H. pylori* agar, modified media (Pronadisa, Spain) supplemented with 7% defibrinated sheep blood (Bahar-Afshan, Iran) and incubated for 5–7 days at 37°C in microaerophilic conditions. Detection of *H. pylori* was based on clonal appearance, Gram staining, and biochemical tests (catalase, oxidase, and urease positive). All isolates were stored in BHI media with the addition of 20%–25% glycerol and 10% fetal bovine serum at -70°C. Then, *H. pylori* strains were subcultured in Brucella Broth media (Merck, Darmstadt, Germany) supplemented with 10% of fecal calf serum (FCS). The broth media were incubated at 37°C for 72 h in microaerophilic conditions. For inhibition of clump formation, shaking was performed constantly during incubation. Isolation of *H. pylori*-derived OMVs was performed from the supernatant of broth media. After 72 h of incubation, 500 ml of media was centrifuged at 15,000 g for 15 min at 4°C to pellet bacteria. Then, the supernatant was filtered through 0.45-µm routine bacteriology filters (Corning, USA). After that, OMVs were isolated by ultra-centrifugation (Beckman, CA, USA) at 150,000 g for 3 h at 4°C. Isolated OMVs were washed in 10 ml sterile distilled water and stored at -80°C for the next steps. The morphology and estimated size of OMVs were confirmed by scanning electron microscopy (SEM) and dynamic scattering light (DLS) techniques.

### Cell Cultures and Treatments

A hepatocyte cell line (AML-12) was obtained from Histogenotech Company, and a hepatic stellate cell line (LX2) was kindly gifted from Professor Scott L. Friedman (Mount Sinai School of Medicine, New York, USA). The LX2 cell line was grown in Dulbecco’s modified Eagle medium (DMEM) (Gibco-Invitrogen, Waltham, MA, USA) supplemented with 10% v/v fetal bovine serum (FBS) (Gibco-Invitrogen, USA) and 1% penicillin–streptomycin (Gibco-Invitrogen, USA), and both cells were kept at 37°C under the presence of 5% CO_2_ and 95% humidified atmosphere. For hepatocyte-OMV coinfection, hepatocytes were cultured in a 25-ml normal flask and cells were kept at 37°C with 5% CO_2_. After 24 h, based on our previous observations, 10 μg/ml of outer membrane vesicles of *H. pylori* was added to the cells. In the next 24 h, cells were washed three times to remove extracellular OMVs.

### Exosome Isolation and Characterization

Hepatocyte-OMV co-incubated cells were adopted to FBS-free medium due to the presence of exosomes in FBS (Gibco-Invitrogen, USA). The FBS concentrations were reduced from 10% to 5% over 5 days in culture medium. After stabilizing cells in serum media, the exosomes were isolated from cells using Exocib kit (Sibzistfan, Iran) based on the manufacturer’s instruction. Briefly, cell debris was removed by centrifugation at 300 g for 10 min and then samples were filtered through a 0.22-µm filter (Corning, USA). Then, CD63 and CD9 markers were used to confirm the presence of exosomes by Western blotting. Exosomes were stored at –80C for the next steps.

### Exosome Coculturing With the LX2 Cell Line

In order to investigate the effects of infected hepatocyte-derived exosomes on hepatic stellate cells, the LX2 cell line was treated with exosomes. LX2 cells were seeded in six-chamber plates and co-incubated with 75 μg/ml concentration of exosomes, and cells were incubated for 48 h at 37°C in the presence of 5% CO_2_.

### RNA Extraction, cDNA Synthesis, and qPCR

Total RNA was extracted from treated cells using the RNA extraction kit according to the manufacturer’s instruction (Favorgen, Taiwan). RNAs were reverse transcribed to cDNA using the commercial cDNA synthesis kit based on the manufacturer’s instruction (Thermo Scientific, Waltham, MA, USA). As shown in **Table 1**, primers were designed for targeted genes using Primer3 software. The real-time PCR technique was performed to investigate the gene expression levels. Briefly, 10 μl of Qmaster Mix (2×) with SYBR Green, 6 μl of water, 1 μl of forward primer, 1 μl of reverse primer, and 2 μl of template were used. The amplification process was performed using Rotor-Gene Q (Applied Biosystems, Warrington, UK).

**Table 1:** Primers used for real-time PCR assay in this study.

Gene	Sequences	Product size (bp)	Reference
β-Catenin	F-GGGTAGGGTAAATCAGTAAGAGGT R-GCATCGTATCACAGCAGGTT	261	32
E-cadherin	F-TGCTCTTGCTGTTTCTTCGG R-CTTCTCCGCCTCCTTCTTC	280	32
TIMP1	F-CACTATGCCGCGCTCTTTC R-TCCTGGAAGGTAAACTCTGGAT	310	32
Vimentin	F-CCAGGCAAAGCAGGAGTC R-CGAAGGTGACGAGCCATT	426	32
α-SMA	F-AGACGGGAATCCTGTGAAGC R-TGTCCCATTCCCACCATCAC	314	32
GAPDH	F-GAAGGTGAAGGTCGGAGTCA R-AATGAAGGGGTCATTGATCA	245	32

### Western Blotting for Protein Expression Levels

In order to confirm the protein expression, Western blotting was performed. Briefly, the protein contents of the cells were extracted using RIPA buffer (Cell Signalling Technology, Germany). The protein concentrations of samples were determined using a BCA protein assay kit. The 4× protein sample dye was added into the samples, and the mixtures were boiled at 99°C for 5 min before gel loading. Thirty micrograms of exosome samples was loaded to each lane and separated onto 10% sodium dodecyl sulfate–polyacrylamide gel electrophoresis (SDS-PAGE) gels. In the next step, proteins were transferred onto PVDF in transfer buffer including 192 mM glycine, 10% methanol, and 25 mM Tris, pH = 8.2. The membrane was blocked with 4% non-fat milk powder in phosphate-buffered saline (PBS)–0.05% Tween for 2 h. The primary antibodies were added in blocking buffer and incubated at 4°C overnight (1:2,000 dilution, Cell Signaling Technology, Danvers, MA, USA), according to the manufacturer’s instructions. The membranes were incubated with primary antibodies against rabbit anti-CD63, CD9, vimentin, TIMP1, α-SMA, β-catenin, and E-cadherin (all obtained from Cell Signaling Technology Company). The equal protein lane loading was used as standard, utilizing a monoclonal antibody against the GAPDH protein (Sigma-Aldrich, St. Louis, MO, USA). The membranes were washed three times with PBS-T [0.1% (v/v) Triton X-100] buffer for 30 min and probed with horseradish peroxidase (HRP)-conjugated secondary antibodies for 2 h. When the washing process with PBS-T buffer was performed, the protein bands were visualized using the Odyssey Infrared Imaging System (LI-COR).

### Statistical Analysis

Statistical analysis was performed with GraphPad Prism software version 8 (GraphPad Software, Inc., La Jolla, CA, USA). Variations between different groups were calculated applying one-way analysis of variance (ANOVA) and t-test for comparisons between each group. Results are presented as the average ± standard error of the mean (SEM) of at least three experiments, unless otherwise stated. Differences were considered statistically significant when P < 0.05; *P < 0.05, **P < 0.01, ***P < 0.001, and ****P < 0.0001.

## Results

### Four Different *H. pylori* Strains and Their OMV Characterization

**Figure 1:**
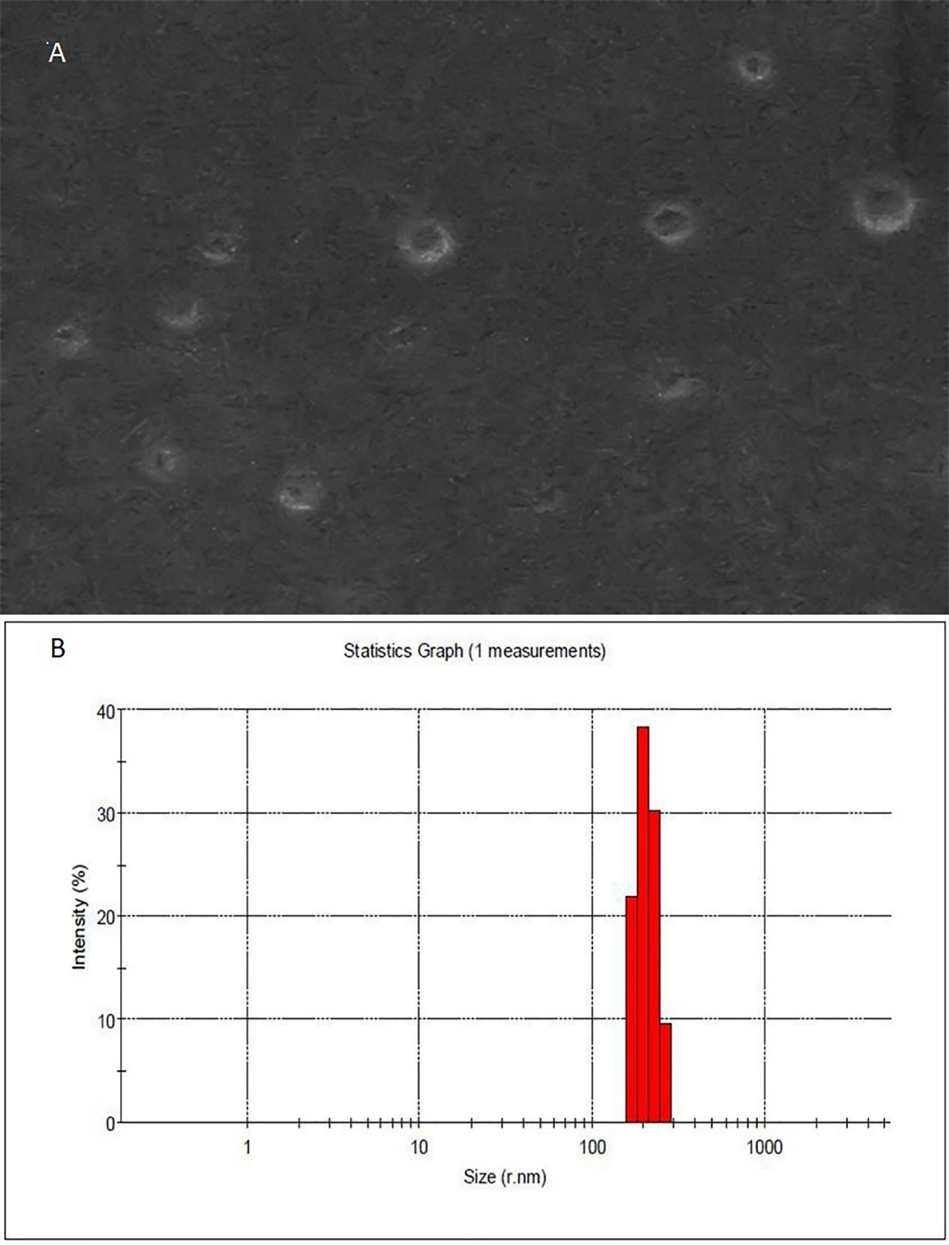
Morphological characterization of *H. pylori*-derived OMVs. **(A)** Scanning electron microscopic (SEM) image of OMVs showed the spherical and double-layered vesicles in different sizes. **(B)** Physicochemical characteristics of *H. pylori*-derived OMVs based on DLS. Size distribution is determined based on the intensity of OMVs in the ultracentrifugation technique. DLS confirmed nano-sized OMVs in a range of about 130–300 nm, peaked at 200 nm.

Four different *H. pylori* isolates were obtained from the Medical Microbiology Research Center, Qazvin, Iran. Based on pathology and clinical observation, three of them were isolated from patients with chronic gastritis and one other strain was from patients suffering from peptic ulcer disease. As shown in **Figure 1**, isolated OMVs were characterized by scanning electron microscopy and dynamic light scattering; more than 90% of OMVs have sizes ranging between 50 and 500 nm. Also, scanning electron microscopy results confirmed a round-shaped morphological structure of OMVs.

### Exosome Isolation From *H. pylori* OMV-Treated Hepatocyte Cells

**Figure 2:**
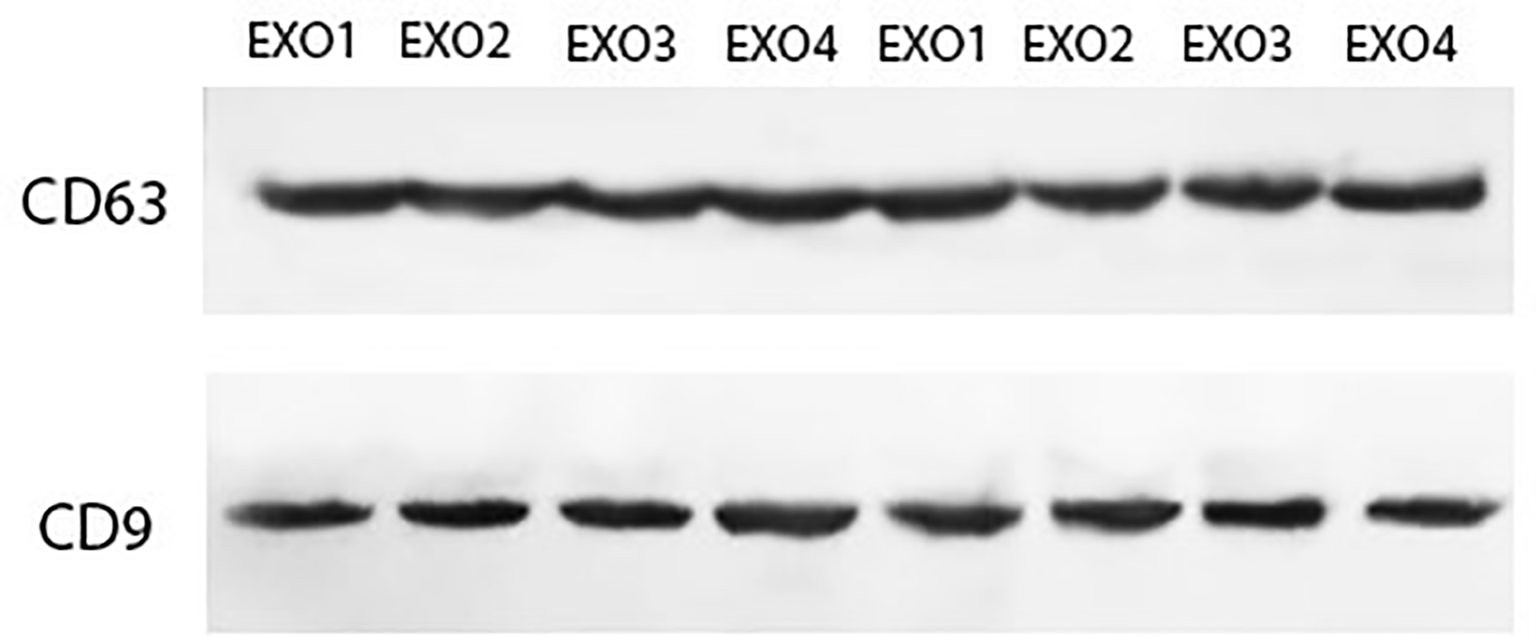
Characterization of hepatocyte-derived exosomes using Western blotting. Hepatocyte cell-derived exosomes were analyzed by Western blotting using antibodies against exosomal markers (CD9 and CD63).

*H. pylori* OMVs enter human cells to alter their pro-inflammatory response and also their exosome contents. In this study, OMV-co-incubated hepatocyte-derived exosomes were isolated to determine their effects on HSCs. In the current study, we used immunoblotting for exosome detection. As shown in **Figure 2**, both CD9 and CD63 was detected which are the most known exosomal markers.

### Hepatocyte-Derived Exosomes and Their Effects on Hepatic Stellate Cells

**Figure 3:**
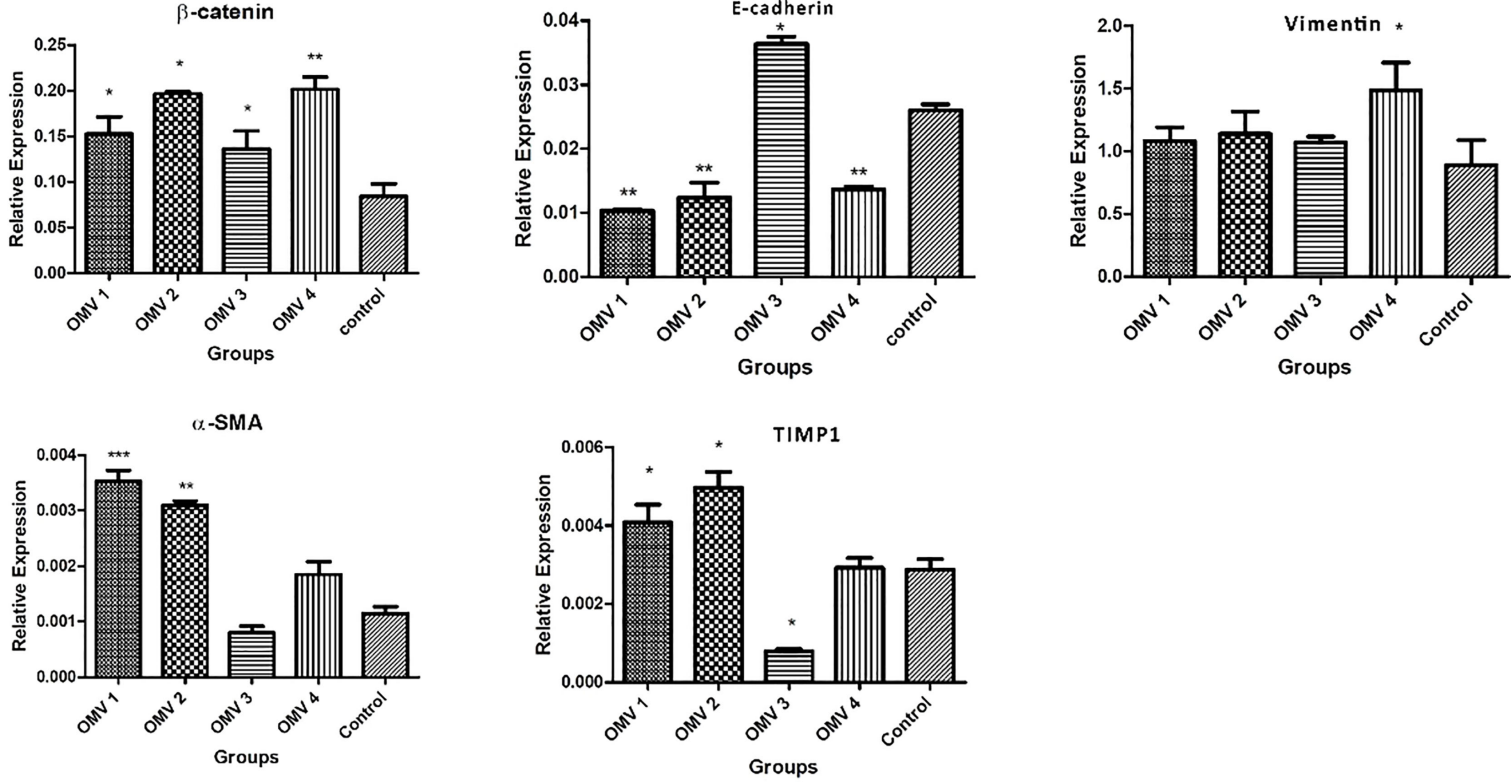
Gene expression of liver fibrosis markers in LX-2 cells upon treatment with 10 µg/ml of *H. pylori* OMVs. Relative gene expression of fibrosis markers (vimentin, β-catenin, TIMP1, and α-SMA) was markedly increased after treatment of LX-2 cells with 10 µg/ml of *H. pylori* OMVs. mRNA level of E-cadherin was decreased in LX-2 cells treated with 10 µg/ml of *H. pylori* OMVs. Data presented as means ± standard error (SEM) for three independent experiments. Data are shown as the mean ± SEM. *P < 0.05, **P < 0.01, ***P < 0.001 and by *post-hoc* one-way ANOVA statistical analysis.

**Figure 4:**
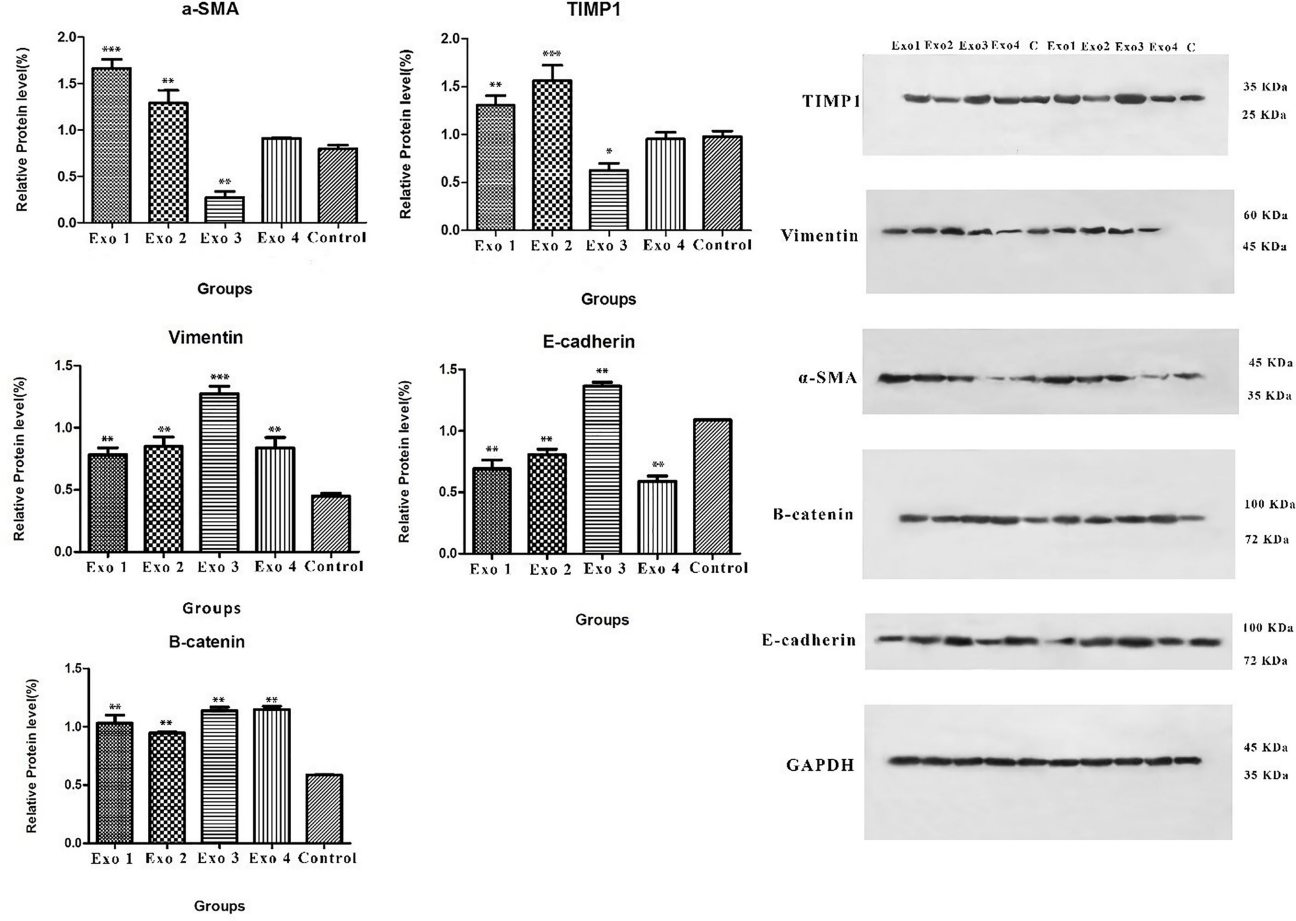
Immunoblotting result of vimentin, β-catenin, TIMP1, α-SMA and E-cadherin protein expressions. As it shown vimentin, β-catenin, TIMP1, α-SMA protein expression of HSCs increased after treatment with infected hepatocytes-derived exosomes. Data presented as means ± standard error (SEM) for three independent experiments. Data are shown as the mean ± SEM. *P < 0.05, **P < 0.01, and ***P < 0.001 by post hoc one-way ANOVA statistical analysis. Scale bar: 20 µm, magnification 200X.

In the next step, we examined the effects of *H. pylori* OMV-contaminated hepatocyte-derived exosomes on the development of hepatic stellate cell activation and fibrosis pathways. After exposing HSCs to exosomes, several activating and fibrosis-related factors were determined including α-SMA, TIMP1, vimentin, e-cadherin, and β-catenin. Exosomes from OMV-contaminated hepatocytes demonstrate an amplified expression of HSC activation markers (α-SMA and TIPM1) and fibrosis markers (vimentin, e-cadherin, and β-catenin) compared with non-treated hepatic stellate cells. The expression analysis was done with both real-time PCR and Western blotting analysis as shown in **Figures 3** and **4**. Immunoblotting results were similar to real-time PCR results, showing overexpression of HSC activation markers (α-SMA and TIPM1) and fibrosis markers (vimentin, e-cadherin, and β-catenin) compared with non-treated hepatic stellate cells.

## Discussion

Investigating the accurate mechanism of non-alcoholic fatty liver disease (NAFLD) development is a critical key factor for the diagnosis and correct treatment of this disease. One of the most important factors in NAFLD is pathogenic microbes, their pathogenesis, and virulence factors. There is strong evidence that *H. pylori* infection has been associated with adverse effects throughout the gastrointestinal tract; there has been speculation regarding the possible association of *H. pylori* infection with the development of NAFLD, which is the hepatic component of metabolic syndrome. There is evidence of an association initially centered on the discovery of the presence of anti-*H. pylori* immunoglobulin G (IgG) in patients with NAFLD.

In this study, we found that adverse effects of *H. pylori* OMVs on hepatocytes and exosomes derived from treated cells showed a potential role in HSC activation and liver fibrosis induction. In the recent decades, outer membrane vesicles have been introduced as an important delivery system of bacterial components and their potent virulence factors. Lee et al. by working on the *H. pylori* 26695 strain showed that Urease A was translocated into gastric epithelial AGS cells through outer membrane vesicles (OMVs) and then targeted the nuclei of AGS cells (Lee et al., 2015). Lekmeechai et al. showed the katG presence in *H. pylori* OMV which confirmed OMV-dependent KatA activity resulting in neutralization of reactive oxygen species (ROS) and helping bacteria evade from oxidative damage (Lekmeechai et al., 2018). Also, the roles of *H. pylori* and *H. pylori*-derived OMVs in extra-gastric disease have been reported in different studies; Lu et al. in 2018 showed the important relation between *H. pylori* and metabolic abnormality among patients suffering from NAFLD (Lu et al., 2018). Their study included 1,867 individuals, and their blood parameters such as ALT, fasting blood sugar, albumin, and uric acid were examined in which there were significant differences in ALT and HDL-C between the study participants with and without *H. pylori* infection. More importantly, Kang et al. demonstrate that there is a strong association between *H. pylori* CagA-negative strains and NAFLD. CagA-negative *H. pylori* infection could be an independent predictor of NAFLD in the US population which was worked on 5,404 participants (Kang et al., 2018). Chen et al., by working on an elderly population in China, showed that there is a complex biological association between *H. pylori* infection and NAFLD. Their clinical results demonstrated that the *H. pylori* infection rate in the NAFLD group was significantly higher than that in the group without NAFLD (Chen et al., 2017). Most recently, Abo-Amer et al. by working on 646 patients demonstrated the *H. pylori* is an independent risk factor for liver steatosis progression (Abo-Amer et al., 2020). Their report showed that NAFLD, ALT, AST, hepatomegaly, hypertension, and fasting blood sugar were significantly higher in the *H. pylori*-positive group than in the *H. pylori*-negative group. On the other hand, some studies have found no association between *H. pylori* and NAFLD (Okushin et al., 2015; Cai et al., 2018; Lu et al., 2018). Our results confirmed the adverse effects of *H. pylori*-derived OMVs on hepatocytes; however, more studies needed confirmation on the *H. pylori* association with liver disease especially NAFLD.

Exosomes are small extracellular vesicles that are involved in cell-to-cell communication. The role of exosomes has been studied in different liver diseases such as alcoholic liver disease, NAFLD, liver fibrosis, liver cirrhosis, and hepatocellular carcinoma. In the current study, we showed that exosomes derived from hepatocytes coinfected with *H. pylori* OMVs have a role in HSC activation and hepatic fibrosis marker expression; similar to our findings, Pavero et al. in different studies revealed that hepatocytes generated membrane-bound vesicles, microparticles, in response to conditions that mimicked the lipid accumulation that occurs in the liver in NAFLD; also, hepatocyte-derived exosomes which are released during lipotoxicity are efficiently engulfed by HSCs resulting in their activation and the significant upregulation of their profibrogenic genes (Povero et al., 2015). Also, there are a mass of studies confirming hepatocyte-derived exosome effects on HSC activation *via* miRNA profiles and there are reports that demonstrate the effects of microbial infections in modifying exosome contents for infection progression. Thus, exosomes are shown as an important factor in cell-to-cell communication and *H. pylori*-derived OMV potential in modifying hepatocyte-derived exosome contents and their effects on HSC activation.

Liver fibrosis is a major consequence of chronic liver disease including NAFLD with poor prognosis (Rosso et al., 2020). HSCs play a key role in the fibrosis process, because in chronic liver damage, they transdifferentiate from a “quiescent” to an “activated” phenotype. α-SMA and vimentin are known as highlighted markers of activated HSCs; however, TIMP-1, β-catenin, and e-cadherin are involved (Zhang et al., 2017). Our investigation of exosomes and their role in HSC activation have confirmed exosomes as key mediators in HSC–hepatocyte connections. In the current study, we found that exosomes from infected hepatocytes with *H. pylori* OMVs promote HSC activation *via* elevated β-catenin, α-SMA, and TIMP1 protein levels and downregulated e-cadherin proteins which revealed the hepatic fibrosis. Similar to our work, Chi-Cheng et al. by working on 126 gastric ulcer patients showed that *H. pylori*-infected gastric ulcers had even higher MMP-7, MMP-9, and TIMP-1 expressions in epithelial cells compared to NSAID-related gastric ulcers (Cheng et al., 2012). Krzysiek-Maczka et al. reported that CagA- and VacA-positive strains of *H. pylori* induce differentiation of normal fibroblasts into cancer-associated fibroblasts which have overexpressed α-SMA and vimentin mRNA levels (Krzysiek-Maczka et al., 2018).

In conclusion, in recent years because of the high prevalence of *H. pylori* among individuals, concerns about *H. pylori* extra-gastric diseases especially liver disease have been raised. However, due to lack of sufficient studies in this regard, the mechanisms are not clear yet. Based on our results, the OMVs showed adverse effects on hepatocyte-derived exosome contents, which may contribute to OMV pathogenesis. Also, we found that modifying exosomes purified from OMV-infected hepatocytes could trigger the activation of HSC. Overall, our study supported the idea that *H. pylori*-derived OMVs are involved in extra-gastrointestinal disorder pathogenesis. More studies are recommended to reveal the exact and precise mechanisms of *H. pylori* extra-gastrointestinal manifestation pathogenesis.

## Data Availability Statement

The original contributions presented in the study are included in the article/supplementary material. Further inquiries can be directed to the corresponding authors.

## Ethics Statement

This work does not contain any studies related with human participants or animals. The study was approved by the Qazvin University of Medical Sciences (Project No. IR.QUMS.REC.1400.379).

## Author Contributions

Cellular and molecular experiments and manuscript drafting: MZ; immunoblotting: MJ and AP; statistical analyses: SS and NH; project supervision and management: SB, RS, and AP. Immunoblotting analysis: HD. All authors contributed to the article and approved the submitted version.

## Funding

This work was supported by the Medical Microbiology Research Center (grant 40495).

## Conflict of Interest

The authors declare that the research was conducted in the absence of any commercial or financial relationships that could be construed as a potential conflict of interest.

## Publisher’s Note

All claims expressed in this article are solely those of the authors and do not necessarily represent those of their affiliated organizations, or those of the publisher, the editors and the reviewers. Any product that may be evaluated in this article, or claim that may be made by its manufacturer, is not guaranteed or endorsed by the publisher.
